# Modeling and Validation of Environmental Suitability for Schistosomiasis Transmission Using Remote Sensing

**DOI:** 10.1371/journal.pntd.0004217

**Published:** 2015-11-20

**Authors:** Yvonne Walz, Martin Wegmann, Stefan Dech, Penelope Vounatsou, Jean-Noël Poda, Eliézer K. N'Goran, Jürg Utzinger, Giovanna Raso

**Affiliations:** 1 Department of Remote Sensing, Institute for Geography and Geology, University of Würzburg, Würzburg, Germany; 2 United Nations University–Institute for Environment and Human Security, Bonn, Germany; 3 German Remote Sensing Data Centre, German Aerospace Centre, Oberpfaffenhofen, Germany; 4 Department of Epidemiology and Public Health, Swiss Tropical and Public Health Institute, Basel, Switzerland; 5 University of Basel, Basel, Switzerland; 6 Institut de Recherche en Sciences de la Santé, Ouagadougou, Burkina Faso; 7 Unité de Formation et de Recherche Biosciences, Université Félix Houphouët-Boigny, Abidjan, Côte d’Ivoire; 8 Centre Suisse de Recherches Scientifiques en Côte d’Ivoire, Abidjan, Côte d’Ivoire; Emory University, UNITED STATES

## Abstract

**Background:**

Schistosomiasis is the most widespread water-based disease in sub-Saharan Africa. Transmission is governed by the spatial distribution of specific freshwater snails that act as intermediate hosts and human water contact patterns. Remote sensing data have been utilized for spatially explicit risk profiling of schistosomiasis. We investigated the potential of remote sensing to characterize habitat conditions of parasite and intermediate host snails and discuss the relevance for public health.

**Methodology:**

We employed high-resolution remote sensing data, environmental field measurements, and ecological data to model environmental suitability for schistosomiasis-related parasite and snail species. The model was developed for Burkina Faso using a habitat suitability index (HSI). The plausibility of remote sensing habitat variables was validated using field measurements. The established model was transferred to different ecological settings in Côte d’Ivoire and validated against readily available survey data from school-aged children.

**Principal Findings:**

Environmental suitability for schistosomiasis transmission was spatially delineated and quantified by seven habitat variables derived from remote sensing data. The strengths and weaknesses highlighted by the plausibility analysis showed that temporal dynamic water and vegetation measures were particularly useful to model parasite and snail habitat suitability, whereas the measurement of water surface temperature and topographic variables did not perform appropriately. The transferability of the model showed significant relations between the HSI and infection prevalence in study sites of Côte d’Ivoire.

**Conclusions/Significance:**

A predictive map of environmental suitability for schistosomiasis transmission can support measures to gain and sustain control. This is particularly relevant as emphasis is shifting from morbidity control to interrupting transmission. Further validation of our mechanistic model needs to be complemented by field data of parasite- and snail-related fitness. Our model provides a useful tool to monitor the development of new hotspots of potential schistosomiasis transmission based on regularly updated remote sensing data.

## Author Summary

Schistosomiasis is a parasitic worm infection that is widespread in sub-Saharan Africa where people get in contact with open freshwater bodies. For many years, the strategy to control schistosomiasis was to prevent morbidity through deworming of school-aged children. Recently, transmission control has gained interest, which requires information where and when exactly transmission occurs. We investigated the potential of high-resolution remote sensing data to delineate potential transmission sites of schistosomiasis. Additionally, we characterized the habitat suitability for parasites and snails that are implicated in the schistosomiasis life cycle. Based on environmental field measurements in Burkina Faso and ecological data from the literature, functions of relative suitability were derived to determine the ecological relationship between the environment and snail and parasite fitness. These functions were employed to model the habitat suitability by using remote sensing variables that are aggregated to a habitat suitability index. We found that temporal dynamic of water bodies is one of the most relevant variables. Less relevant were topographic drainage lines. Our model also revealed significant relations with disease prevalence in different ecological zones of Côte d’Ivoire, and thus provides a useful tool to monitor new hotspots of disease transmission based on regularly updated remote sensing data.

## Introduction

Schistosomiasis is a neglected tropical disease caused by blood flukes of the genus *Schistosoma*. From a public health perspective, schistosomiasis is considered the most important water-based disease. Indeed, data from mid-2003 suggest that 779 million people were at risk of schistosomiasis with more than 200 million people infected, predominantly in sub-Saharan Africa [1]. An infection with schistosomes depends on the spatial and temporal distribution of specific freshwater snails that act as intermediate hosts and are the prerequisite that a *Schistosoma* parasite reaches the development stage to infect humans. The global strategy endorsed by the World Health Organization to control schistosomiasis is the large-scale administration of the antischistosomal drug praziquantel to at-risk populations to prevent morbidity [2,3]. The sustainability of this control strategy has been challenged, as there is rapid re-infection after deworming [4–6]. In recent years, a shift occurred from morbidity control to transmission control and local elimination, and hence, there is a stronger focus on intermediate host snails and transmission sites, along with primary prevention tailored to specific social-ecological systems [7–9].

The use of satellite remote sensing data and techniques for risk profiling of environment-related diseases, including schistosomiasis, has increased considerably over the past 30 years [10]. In general, spatial and temporal properties of various remote sensing data and variables are investigated in relation to specific diseases or disease agents, such as intermediate hosts, parasites, or vectors for selected geographic regions of the world with the overall aim to support disease control and prevention strategies [10–17]. The standard approach for schistosomiasis risk profiling using remote sensing data presented in the literature is to relate the measure of *Schistosoma* infection prevalence–predominantly surveyed and geolocated at schools–to remote sensing measurements to model and spatially predict the risk of infection [16,18–21]. However, from a conceptual point of view, the application of remote sensing data aims at characterizing environmental conditions of potential disease transmission sites, which are in many cases spatially disjunct from the school location where epidemiological surveys are usually being conducted [21]. Few studies used remote sensing data to specifically characterize biophysical features of habitats in relation to snail prevalence, such as climatic variables in sub-Saharan Africa [22–24], and flooding and vegetation coverage in the People’s Republic of China [25–27].

Specific habitat requirements of intermediate host snails are governed by environmental factors [28,29]. Beside the fact that intermediate host snails need an aquatic environment, it has been shown that certain conditions, such as water temperature, water flow velocity, and vegetation coverage, affect snail metabolism and fitness with consequences on species presence or absence [30–35]. A literature review on abiotic factors in relation to snail or parasite fitness revealed conditions that determine the abiotic environmental niche of a species and thus the suitability of a habitat as potential disease transmission site [36,37]. The habitat suitability index (HSI) is a methodological approach that allows to model, integrate, and summarize modeled environmental preferences, limits of tolerance, and behaviors of organisms in general. This methodological procedure was originally developed by the United States Fish and Wildlife Service (USFWS) to estimate the capacity of a habitat to support a species and quantify effects of land management alternatives on species habitats [38]. Thus far, USFWS has developed an HSI in over 150 species-specific models with the objective to support informed decision-making with respect to land management and species conservation [39]. Additionally, the HSI has been rigorously investigated for marine species [40,41], wildlife [42–44], and plant species [45–47], however, not yet for environment-related diseases such as schistosomiasis. Only few HSI models have been validated in a comprehensive manner [48], perhaps explained by the unavailability of adequate data to support validation [49]. It has been argued that HSI models belong to the most influential management tools and provide unique platforms to further explore species-environment relationships [50]. The key contribution of HSI models lies in quantifying both the quality and quantity of available habitats for selected species and is useful for monitoring dynamic habitat conditions over time, if current environmental data provide this information [51].

We investigated the potential of high-resolution remote sensing data to model environmental suitability for the transmission of schistosomiasis by means of a deductive mechanistic HSI modeling approach. We discuss strengths and weaknesses of remote sensing data for modeling environmental suitability, placing emphasis on (i) the spatial delineation of environmental conditions where disease transmission can potentially occur; (ii) quantitative prediction of environmental suitability within potential transmission sites; and (iii) transferability of an established model to different ecosystems. To support sustainable interruption of schistosomiasis transmission, spatial predictions of environmental suitability based on remote sensing data are required to expand the modeling perspective from school-located assessments to transmission site characteristics.

## Methods

### Ethics Statement

We present a secondary analysis with data derived from previously published studies that had been approved by the respective institutional review boards and national ethics committees; study site Ouagadougou [52,53], study site Man [54,55], and study site Taabo [56,57]. The studies were conducted according to national and international guidelines. All data received for the current analyses were anonymized.

### Geography, Epidemiology, and Disease Control in the Study Areas

**Fig 1:**
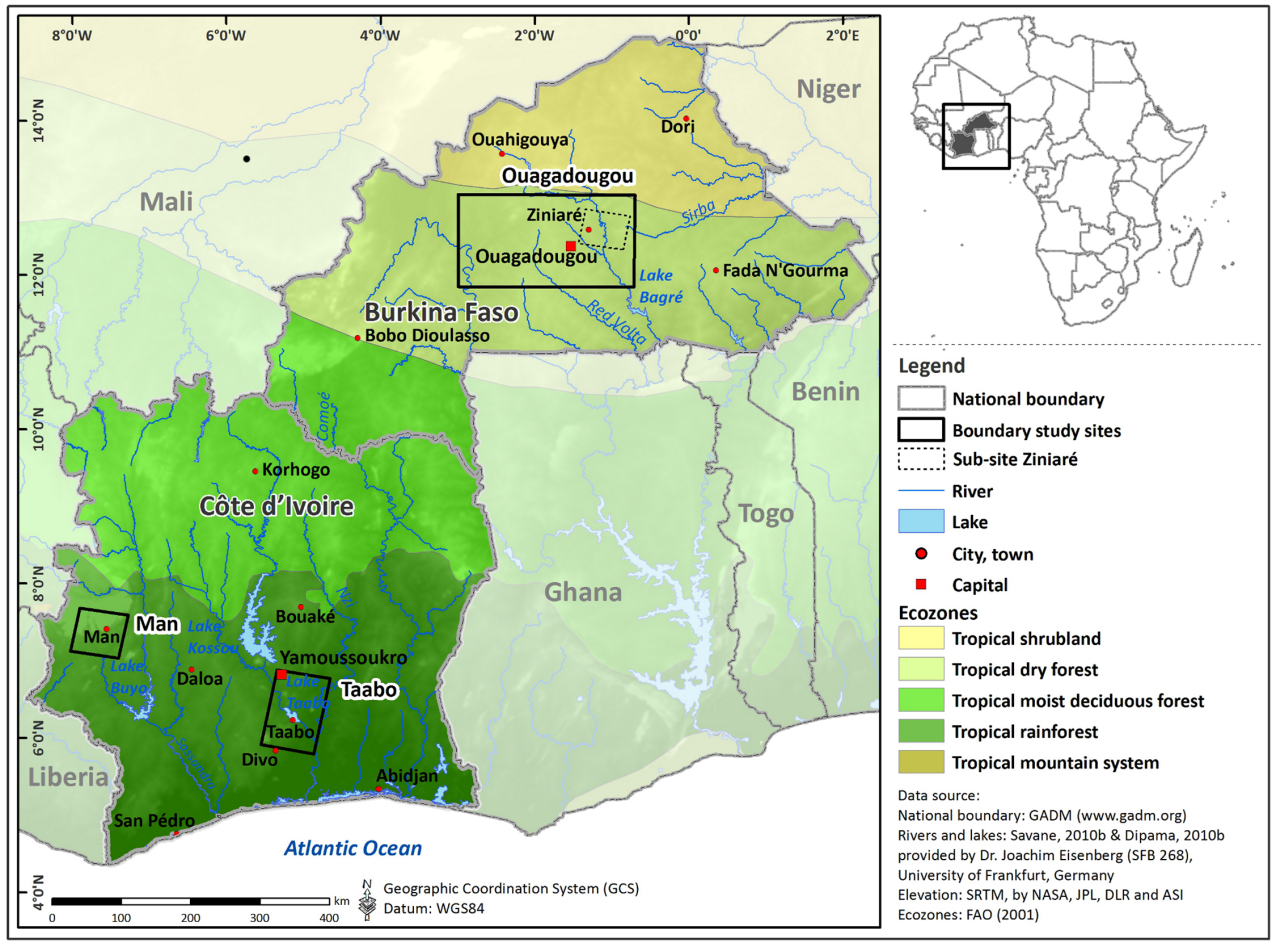
Map of Burkina Faso and Côte d’Ivoire in West Africa, illustrating the three study sites.

The study areas comprise three sites: (i) a large area around Ouagadougou (32,826 km^2^), the capital of Burkina Faso with a small sub-site around the city Ziniaré (3,155 km^2^); (ii) an area in the Man region (4,381 km^2^), located in the western part of Côte d’Ivoire; and (iii) a site around Taabo (8,476 km^2^) in south-central Côte d’Ivoire. The three sites represent a transect of ecozones from dry savannah in the North to tropical rainforest in the South, including flat and mountainous regions (Fig 1). The characteristic diurnal climate shows an annual precipitation gradient increasing from North (Ouagadougou: approximately 790 mm/year) to South (Taabo: approximately 1,230 mm/year and Man: approximately 1,630 mm/year) with mean temperatures above 25°C in all sites [58]. In Burkina Faso, the river network is subdivided by dammed lakes that provide surface water for manifold usage, especially during the dry season when rivers dry out temporarily. These man-made lakes strongly modify the river hydraulic conditions, resulting in reduced water flow velocity. Additionally, ponds are formed where rainwater fills topographic depressions. These play a crucial role for pastoral life and the transmission of schistosomiasis, especially where they constitute the only major water source [59,60].

Schistosomiasis is endemic in all three sites with moderate to high transmission rates at the time the data were collected [61]. The spatial distribution of schistosomiasis shows a typical focal pattern with prevalences at the unit of the school/village ranging between 0% and 100% [62]. While *Schistosoma haematobium* is the predominant species in Ouagadougou and Taabo, *S*. *mansoni* predominates in the Man region [63]. The main intermediate host snails are *Bulinus truncatus*, *Bu*. *globosus*, *Bu*. *senegalensis*, and *Bu*. *forskalii* for *S*. *haematobium*, and *Biomphalaria pfeifferi* for *S*. *mansoni* [64,65]. Based on snail surveys carried out in the 1980s and 1990s in Burkina Faso [64], 41% of intermediate host snails were found in small reservoirs, 34% in rivers, 20% in temporary ponds, 3% in irrigation channels, and 2% in natural lakes. These observations reveal the importance of small reservoirs with respect to the distribution of schistosomiasis in Burkina Faso [66]. The typical sites for potential disease transmission in Côte d’Ivoire are dam lakes, natural ponds, irrigation canals, and river confluences or bulges, where flow velocities of the rivers are slow, coupled with intense human-water contact patterns [65].

The surveillance and control of schistosomiasis are subject to supervision by national health authorities with the consequence that national borders delineate the respective efforts and applied practices between countries. In Burkina Faso, with the assistance from the Schistosomiasis Control Initiative (SCI) [67], nation-wide control of the disease has been implemented by the following steps: (i) identifying the most heavily infected regions; (ii) training local health staff and teachers; (iii) providing health education to the local population; and (iv) large scale administration of praziquantel to school-aged children. During mass treatment campaigns conducted between 2004 and 2006, more than 6 million school-aged children were treated [68]. In Côte d’Ivoire a national control program has been established in 1998, but, due to limited funding and more than a decade of civil unrest, mass drug administration only became feasible in the recent past [69].

### Remote Sensing Data and Derivation of Environmental Metrics

High-resolution remote sensing data from RapidEye (spatial resolution: 6.5 m) [70] with cloud coverage below 5% were acquired for the study sites in Côte d’Ivoire for January 2011, and for the sub-site Ziniaré at the end of the dry season (February 2010) and the end of the rainy season (October 2010). Additionally, six Landsat Thematic Mapper (TM) (30 m) scenes (paths 194–196, rows 51–52) were acquired for the study site in Ouagadougou during the dry (January and February 2010) and at the end of the rainy season (November and December 2010) from the United States Geological Survey (USGS) Global Visualization Viewer (http://glovis.usgs.gov/). Pre-processing of RapidEye and Landsat data involved the orthorectification, atmospheric, and topographic correction using the atmospheric correction tool ATCOR [71] within the CATENA (*lat*. for “chain”) pre-processing tool of the German Aerospace Centre (DLR) [72]. Clouds have been masked based on thresholds in the visible blue and thermal infrared band of Landsat 5 TM data [73]. Geolocation accuracy of the pre-processed Landsat 5 TM data was within 30 m sub-pixel level in reference to RapidEye data. The Advanced Spaceborne Thermal Emission and Reflection Radiometer (ASTER) global digital elevation model (GDEM) (30 m) data were downloaded from the Japan Space Systems [74] and used as basis for topographic analyses given a vertical root mean square error (RMSE) of 8.68 m [75].

Specific environmental metrics were derived from these remote sensing data to build up our habitat suitability model. A water mask has been derived from RapidEye and Landsat 5 TM imagery by thresholding the normalized difference water index (NDWI) [76] with a cut-off value set to zero, above which the spectral index delineates water. However, in the Man study site, the very small ponds and river lines, which are partly covered by dense riparian vegetation together with the exposure of dark rocks, contributed to misclassification. Hence, in this study area, the water bodies were mapped using a hierarchical procedure, including (i) a supervised classification of water/non-water employing a random forest classification algorithm [77]; (ii) the exclusion of topographic landscape elements improbable for the establishment of water bodies (curvature, slope) calculated from the ASTER GDEM; and (iii) the manual refinement of the water mask by visual inspection of RapidEye data in reference to very high-resolution data with spatial resolution below 2 m available in Google Earth [78]. Water surface temperature was measured by the Landsat 5 TM thermal band with an emissivity threshold of 0.98 [79]. The vegetation measure was derived from the RapidEye- and Landsat-based normalized difference vegetation index (NDVI), which responds to the change in the amount of green biomass and chlorophyll content. Additionally, several environmental metrics were derived from the ASTER GDEM, namely sinks, slope and stream order: topographic sinks that refer to locations where all neighboring values were equal to or greater than the center pixel value in the digital elevation model (DEM). Slope of the terrain was calculated with inclination in degrees, and a stream network of topographic water drainage was derived as a result of flow direction and flow accumulation analysis. The resulting stream network has been ordered according to Strahler [80] based on an accumulation threshold of 18,000 cells.

### Validation Data: Environmental Field Data and Parasitological Data

**Fig 2:**
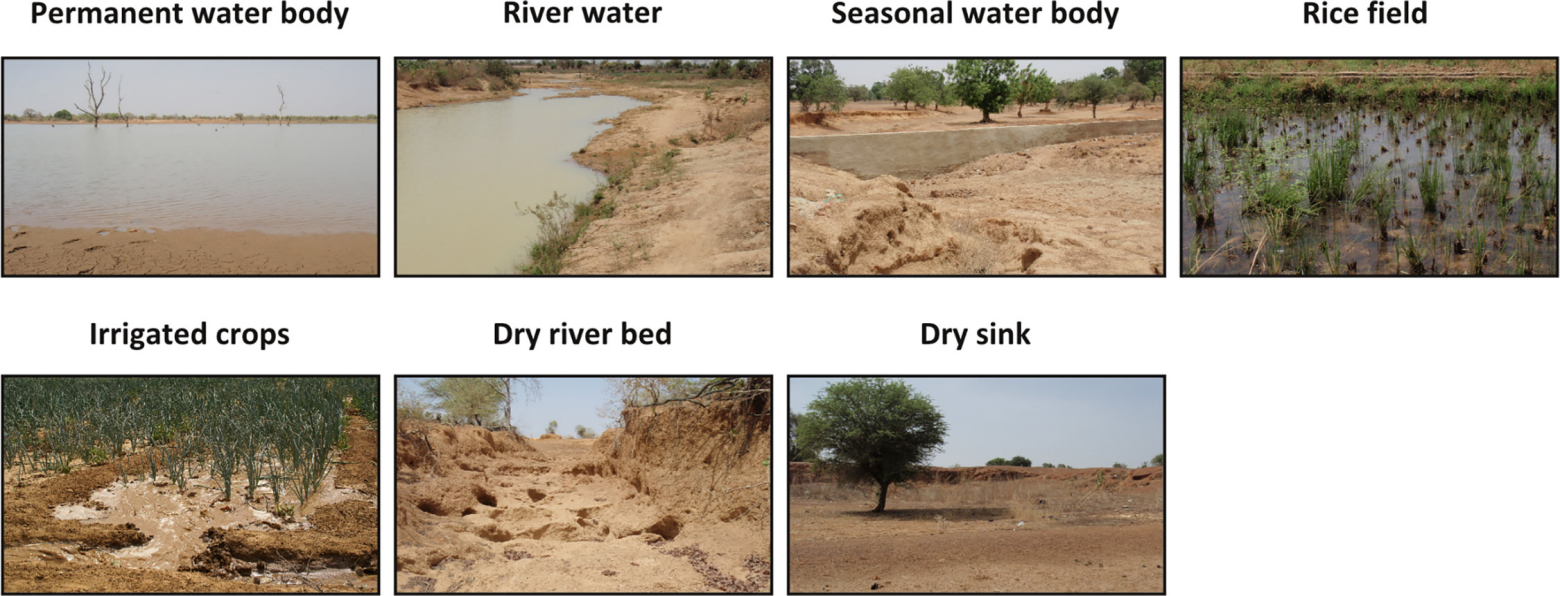
Illustration of field observation at typical schistosomiasis transmission sites.

During field work in the study site in Ouagadougou in March 2011, seven habitat types were identified according to the biotope classification of intermediate host snails in Burkina Faso [64]. A field verification form, adjusted from the standard Food and Agriculture Organization (FAO) Land Cover Classification System (LCCS) [81], guided the local analysis concerning potential disease transmission sites. Most relevant habitat characteristics were recorded at typical schistosomiasis transmission sites and are listed in Table 1 and illustrated in Fig 2. Water persistence, vegetation coverage, and water flow velocity were estimated at the respective sites. Water body temperature was measured using a hand-held digital thermometer at the outer boundary of the water body and revealed temperatures ranging between 26°C (minimum) and 36°C (maximum). Although this measurement is not representative for the entire water body, it indicates a range of water surface temperature in this region. The site-specific estimate of environmental suitability is based on knowledge of habitat preferences of *S*. *haematobium* and *S*. *mansoni* cercariae and *Bulinus* and *Biomphalaria* snails [21].

**Table 1:** Example of field observations at typical schistosomiasis transmission sites. The measurements and estimates in the field were taken in the study site Ziniaré in Burkina Faso during the dry season in March 2011. NA indicates that this measure was not applicable. The typical transmission sites are illustrated in Fig 2. The habitat variable vegetation coverage was assessed based on the standard FAO LCCS form, flow velocity was categorized based on visual inspection, water temperature was measured using a hand-held digital thermometer, and estimation of the overall suitability of the transmission site resulted from knowledge on habitat preferences of species from the literature.

	Permanent water body	Rice field	Irrigated crops	River water	Seasonal water body	Dry river bed	Dry sink
**Vegetation coverage**	NA	75%	60%	NA	30%	20%	20%
**Flow velocity**	Stagnant	Stagnant	Slow flowing	Stagnant	NA	(Fluent: erosive river bed)	NA
**Water tem-perature**	26.2°C	28.5°C	32°C	31°C	NA	NA	NA
**Estimated suitability**	High	High	Moderate	Moderate	Moderate	Low	Low

Parasitological data have been accessed and extracted for the study sites from the Global Neglected Tropical Disease (GNTD) database (http://www.gntd.org) [63,82] with sample sizes of 74 locations for Ouagadougou, 75 for Man, and 38 for Taabo. The GNTD database includes relevant information about the date of survey, description of the survey location and population, number of people examined, diagnostic approach, and prevalence of infection [63]. The final sample of parasitological data used in this study is a result of a preparatory selection procedure that removed surveys conducted before 1980 to respect the maximal life span of the parasites (30 years) [83] and surveys based on diagnostic approaches with a low sensitivity (e.g., direct fecal smear for *S*. *mansoni* infection) from the original database, as described by Schur et al. [84]. The data were used to validate modeled environmental suitability in different ecological zones.

### Development and Composition of Environmental Suitability Model

A mechanistic modeling approach according to Brooks [50] was used to investigate the potential of remote sensing data to assess environmental suitability for the transmission of schistosomiasis. The model was developed for the sub-site Ziniaré in Burkina Faso, where high resolution remote sensing data and environmental field data were available, and tested for its transferability to the study sites Ouagadougou, Man, and Taabo (Fig 1). Specific habitat variables were derived from environmental metrics and parameterized based on functions that describe the relative suitability of each single habitat variable (Table 2). The derivation of functions of relative suitability is depending on the source of information and data provided [38]. The habitat stability is defined as length of water persistence in weeks and was derived from multitemporal RapidEye data from the dry and wet season in the year 2010. The water temperature variable expresses the length of the prepatent period of parasites defined as response function for *S*. *mansoni* and for *S*. *haematobium* [34,35]. Additionally, the relation between water temperature and snail mortality was investigated for snails collected in the field and observed under laboratory conditions. The water flow velocity variable represents the slope measurement of ASTER GDEM data calculated into flow velocity following the Manning’s velocity equation [85] and reflects a critical threshold at 0.3 m/s [86]. The relative suitability of water depth is expressed by the proxy measurement of Euclidean distance from the shoreline, which was calculated from the polyline boundary of the water masks from the dry and wet season. The relative suitability based on vegetation coverage was measured within a 200 m buffer area along detected water sites to capture irrigated agricultural fields (= vegetation suitability) and for water sites that seasonally dried out (= dry season vegetation suitability), as measured in the RapidEye satellite imagery. The NDVI threshold of 0.3 to mark high suitability stems from measurements in dry season RapidEye imagery and corresponds to areas of irrigated agriculture. Stream order resulted in hierarchical levels ranging from order 1 to 7 from a linear interpolation according to a previous study [87]. Sink depth suitability resulted from linear interpolation between the minimum and maximum depth measured in the study area. More details on the derivation of functions of relative suitability used in this study are provided in the Supporting Information. Relative suitability of each habitat variable was scaled between 0 and 1 and was considered highest when most favorable conditions for the coexistence of snail and parasite were given.

**Table 2:** Functions of relative suitability used for modelling environmental suitability based on remote sensing metrics.

Habitat variables [RS data and metrics]	Function of relative suitability	References
**Habitat stability** w (weeks) for development of *S*.* mansoni* (a) and *S*.* haematobium* (b) [RapidEye/Landsat multitemporal water mask]	(a) $f(w)=\left\{ \begin{array}{c} 0\;for\;0<w\leq 4\\ 0.5w-2.5\;for\;5\leq w\leq 7\\ 1\;for\;8\leq w\leq 52 \end{array}\right.$ (b) $f(w)=\left\{ \begin{array}{c} 0\;for\;0<w\leq 3\\ 0.5w-2\;for\;4\leq w\leq 6\\ 1\;for\;7\leq w\leq 52 \end{array}\right.$	[30,33,88]
**Water temperature** T (°C) for *S*.* mansoni* (a), *S*.* haematobium* (b), *Biomphalaria glabrata*(c) and *Bulinus truncatus* (d) [Landsat band 6 thermal emissivity]	(a) $f(T)=\left\{ \begin{array}{c} 0\;for\;T<16\\ -0.003*\left(\frac{268}{T-14.2}-335\right)\;for\;16\leq T\leq 35\\ 0\;for\;35<T \end{array}\right.$ (b) $f(T)=\left\{ \begin{array}{c} 0\;for\;T<17\\ -0.006*\left(\frac{295}{T-15.3}-174\right)\;for\;17\leq T\leq 33\\ 0\;for\;33<T \end{array}\right.$ (c)* $f(T)=\left\{ \begin{array}{c} 0\;for\;T<16\\ -4.095+0.368\;T-0.007\;T^{2}\;for\;16\leq T\leq 35\\ 0\;for\;35<T \end{array}\right.$ (d) $f(T)=\left\{ \begin{array}{c} 0\;for\;T<17\\ -2.350+0.208\;T-0.004\;T^{2}\;for\;17\leq T\leq 33\\ 0\;for\;33<T \end{array}\right.$	[31,32,34,35,89]
**Water flow velocity** calculated from S [ASTER GDEM slope in degrees]	$f(S)=\left\{ \begin{array}{c} -5714.3\;S+1\;for\;0\leq S\leq 0.00014\\ -0.0029\;S+0.2\;for\;S\;>0.00014 \end{array}\right.$	[33,83,86,88]
**Water depth** calculated from distance to shore x (m) [boundary of RapidEye/Landsat multitemporal water mask]	$f(x)=\left\{ \begin{array}{c} -0.0043x+1\;for\;0\leq x\leq 210\\ -0.000056x+0.088\;for\;210\;>x\;\leq 2000\\ 0\;for\;x>2000 \end{array}\right.$	[90]
**Vegetation coverage** V [NDVI derived from RapidEye/Landsat data]	$f(V)=\left\{ \begin{array}{c} 0\;\text{for}\;V<0\\ 3.33\;V\;\text{for}\;0\geq V\leq 0.3\\ 1\;for\;V>0.3 \end{array}\right.$	[30]
**Stream order** (stream) [Topographic drainage lines derived from ASTER GDEM]	*f*(*stream*) = 0.143 *stream for* 0 ≤ *stream* ≤ 7	[87]
**Sink depth** z (m) [ASTER GDEM-based sink depth]	*f*(*z*) = 0.005*z* + 0.11 *for* 1 ≤ *z* ≤ 222	New variable

The derivation of these functions is explained in detail in the Supporting Information.

* This formula was originally derived for *Biomphalaria glabrata* strains in the laboratory. The validity of the formula was also tested in African snail species *B*. *pfeifferi* and *B*. *alexandrina* and agreed very well with the original data [34].

**Fig 3:**
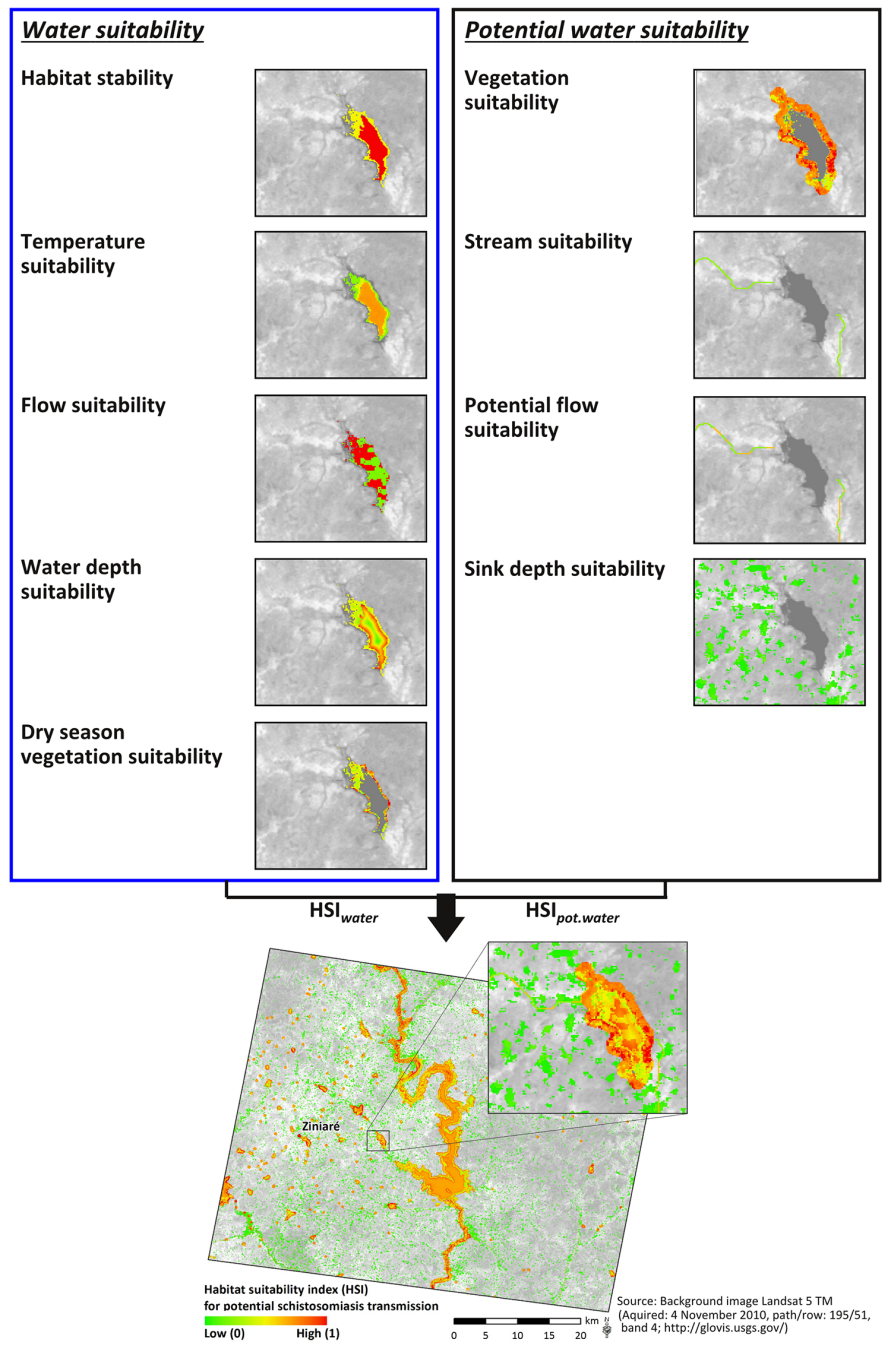
Overview of single habitat variable suitability and the overall HSI. This Figure represents the output of modeled environmental suitability for schistosomiasis transmission at the sub-site of Ziniaré in Burkina Faso for the year 2010, based on which the model was developed.

In a next step, habitat variables were combined to an overall HSI using a multi-criteria decision analysis (MCDA) [91]. In this study, the aggregation of habitat variables to the HSI was conducted using an additive priority function, as follows:

$$HSI=\sum_{i=1}^{m}\;a_{i}f_{i}$$

where *HSI* stands for the habitat suitability index and refers to the global priority of environmental suitability, *m* indicates the number of habitat variables, *a*_*i*_ describes the weighting of habitat variable *i*, and *f*_*i*_ gives the relative suitability of the habitat variable *i* between 0 and 1. Due to the lack of appropriate reference data to calibrate the model, *a*_*i*_ was weighted for each habitat variable by 1/m. The HSI was calculated separately for spatially superimposing habitat variables to capture (i) the relevant suitability at water sites (HSI_*water*_) by aggregating the variables habitat stability, water temperature suitability, water flow suitability, water depth suitability, and dry season vegetation suitability, and (ii) relevant topographic characteristics where water can potentially accumulate (HSI_*pot*.*water*_) by aggregating vegetation suitability within the 200 m buffer zone, stream suitability, potential flow suitability in drainage lines, where no water was detected by remote sensing data and sink depth suitability (Fig 3). The overall HSI value for a study site resulted from the juxtaposition of the HSI_*water*_ and HSI_*pot*.*water*_ derived for the defined spatial units (Fig 3).

### Model Validation

The plausibility of modeled environmental suitability based on remote sensing data was validated in reference to on-site measurements and estimations presented in Table 1. Selected test sites were classified into high (HSI>0.67), moderate (HSI 0.33–0.67) and low (HSI<0.33) environmental suitability. Areas of no environmental suitability for schistosomiasis transmission correspond to regions where water cannot accumulate.

Additionally, the modeled environmental suitability was related to schistosomiasis prevalence based on the assumption that high environmental suitability in the catchment area of the surveyed location would be plausible if the measured prevalence was high and *vice versa*. It was the aim to elaborate whether this hypothesis can be confirmed and whether this locally developed model can be transferred to different ecozones in the Man and Taabo areas of western and south-central parts of Côte d’Ivoire, respectively. The linkage between modeled environmental suitability and schistosomiasis was established by spatially extending the point measurement of infection prevalence by a circular buffer with a radius of 5 km [1,92,93]. Within this buffer region, mean values of habitat variables and the composite HSI were extracted for each surveyed catchment area. The statistical relation between the mean environmental suitability and the measured prevalence was assessed by the Spearman rank correlation coefficient [94]. Given that field data of parasite- and snail-related fitness were not available for this study to directly validate the HSI, disease prevalence data were used instead to validate environmental suitability as prevalence documents the outcome of the disease transmission process in the environment.

## Results

### Remotely Sensed Environmental Suitability

The resulting suitability of each habitat variable for water sites (HSI_*water*_), potential water sites (HSI_*pot*.*water*_), and the resulting HSI for the model development site of Ziniaré are illustrated in Fig 3. At water sites (HSI_*water*_), highest suitability was measured for permanent water bodies and moderate suitability for temporary ones, which dried out during the dry season. Water temperature suitability represented the mean temperature suitability for *S*. *haematobium* and *S*. *mansoni* worms and *Bulinus* and *Biomphalaria* snails calculated from the dry and wet season water surface temperature. Most water bodies showed generally moderate to high temperature suitability for parasites and snails, whereas the riparian regions along the water bodies resulted in moderate to low temperature suitability. The relative suitability of water flow velocity showed that within several small-scale water sites, the suitability of flow velocity appeared to be heterogeneous, ranging from very low to very high. Nonetheless, one would expect stagnant or very slow moving water due to its topographic constitution as dam lake. Relative suitability of dry season vegetation coverage highlights areas of very high suitability, where dense vegetation covers a ground that was flooded during the rainy season corresponding to irrigated agriculture in this region.

At potential water sites (HSI_*pot*.*water*_), the relative suitability of riparian vegetation coverage highlighted areas of irrigated agriculture and dense vegetation coverage as highly suitable, whereas highest suitability was reached when dry season and wet season vegetation coverage were high and irrigation was possible throughout the year. Topographic drainage lines resulted in high suitability at their lowest hierarchical order at the inflow and outflow of the great dam lake in the center of the study site and were modified by the overlaying suitability of flow velocity derived from the topographic slope. The sink habitat variable resulted primarily in very low suitability due to its flat character, whereas deeper sinks were masked due to their coverage with water.

The HSI showed a general discrimination between moderate to high suitability in and around water bodies and low suitability at topographic sinks. Individual water sites performed with variable suitability depending on the location in or around the water site. The zoomed-in part of the image in Fig 3 shows that high suitability pertained to areas at densely vegetated sites in the buffer zone of the water site and in certain areas of the water body, where especially water flow velocity was low. Moderate suitability referred to the littoral zone of permanent water levels, low vegetation cover buffer zones, and certain sectors of the topographic drainage line, whereas potential flow velocity resulted in high suitability. Low suitability resulted predominantly from topographic sinks, low vegetated region of seasonal flooded land, and sparsely vegetated regions in the buffer zone around the water site. Especially densely vegetated zones around permanent water sites resulted in high environmental suitability.

### Plausibility of Modeled Environmental Suitability

The plausibility of modeled environmental suitability in reference to field estimates (Table 1) are summarized and illustrated in Table 3 for the Ziniaré site within Ouagadougou. The measured HSI of 0.6 at the permanent waterbody (Table 3, row 1) was composited by the suitability based on habitat stability (= 1), temperature suitability (= 0.41) resulting from surface temperature of 30°C in the dry season and 35°C in the wet season, flow suitability (= 0.20), and water depth suitability (= 0.81). At this site, the estimated high suitability did not meet the modeled HSI. In contrast, the HSI of 0.55 at the river water (Table 3, row 2) resulted in conformity with the field estimated moderate environmental suitability. The permanent water coverage was correctly detected by remote sensing data. However, the modeled temperature and flow suitability resulted in very low environmental suitability, which does not correspond to the at-site field measurements of stagnant water during the dry season and water temperature of 31°C. At the seasonal waterbody (Table 3, row 3), the HSI was slightly below the field estimated habitat suitability. However, the habitat variables showed plausible suitability.

**Table 3:** Result of modeled suitability of single habitat variables and the HSI in comparison to field-based habitat suitability estimates for water sites and potential water sites.

Type of habitat	Result of habitat variables	HSI value	Estimated habitat suitability at location (see Table 1)
**Permanent water body**	1 (HS); 0.41 (TS); 0.20 (FS); 0.81 (DS)	0.60 (moderate)	High
**River water**	1 (HS); 0 (TS); 0.19 (FS); 1 (DS)	0.55 (moderate)	Moderate
**Seasonal water body**	0.50 (HS); 0 (TS); 0.20 (FS); 0.50 (DS); 0.42 (dVS)	0.32 (low)	Moderate
**Rice field**	0.97 (bVS)	0.97 (high)	High
**Irrigated crops**	0.64 (bVS)	0.64 (moderate)	Moderate
**Dry river bed**	0.55 (bVS)	0.55 (moderate)	Low
**Dry sink**	0.08 (SiS)	0.08 (low)	Low

Abbreviations: bVS = mean vegetation suitability within 200 m buffer zone of water; DS = water depth suitability; dVS = dry season riparian vegetation suitability; FS = water flow suitability; HS = suitability of habitat stability; SiS = sink suitability; TS = water temperature suitability

The HSI of potential water sites (Table 3, rows 4–7) showed that, except for the dry sink test site, the suitability of mean vegetation coverage within a 200 m buffer zone of water was relevant to determine the HSI. The modeled HSI values reflected well the high and moderate suitability estimates for rice and irrigated crop sites, respectively. However, the estimated suitability of rice and irrigated crop sites resulted mainly from the irrigation practice, which varies between a permanently flooded rice field and crops that were irrigated by temporary flooding. This measure was not captured by remote sensing data in this case. The low environmental suitability estimated for the dry sink corresponded well to the modeled HSI of 0.08. However, the dry river bed was only captured due to its position within a 200 m buffer zone of water and resulted in a HSI value of 0.55 as a consequence of vegetation coverage at the site of the river bed. The HSI measure at this site would be expected to result from stream and potential flow suitability. However, this dry river bed was not detected by topographic drainage lines.

### Model Transferability

**Fig 4:**
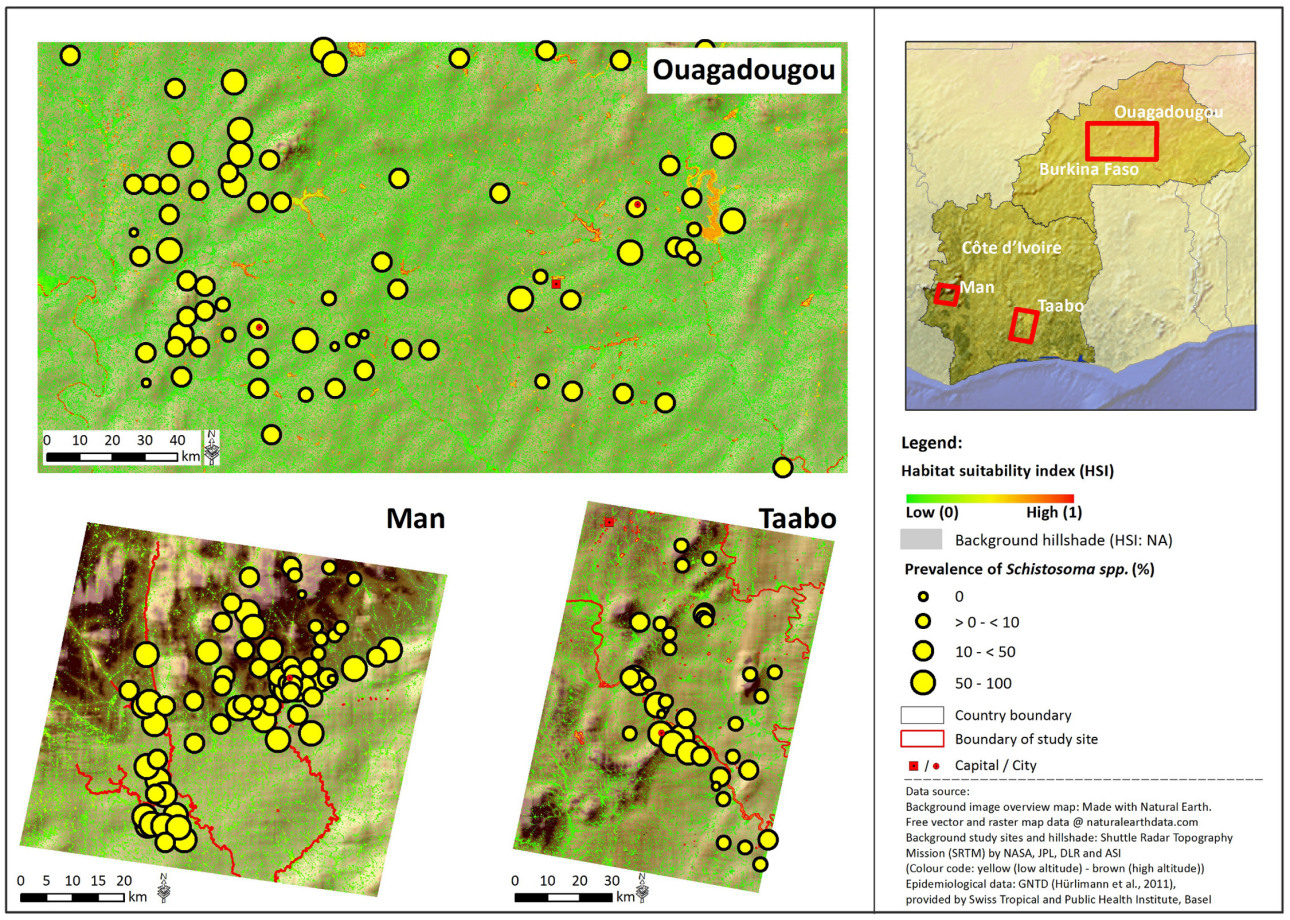
Habitat suitability index (HSI) and epidemiologic measurements at school locations for the three study sites Ouagadougou (Burkina Faso), Man and Taabo (Côte d’Ivoire), West Africa. In Ouagadougou and Taabo, schistosomiasis is mainly caused by *S*. *haematobium*, whereas in Man *S*. *mansoni* is widespread.

The environmental suitability was plausible, as evaluated for the model development site Ziniaré. The HSI model was then applied to remote sensing data covering the larger study site Ouagadougou and the two study sites in Côte d’Ivoire (Man and Taabo). The transferability of the model from Ziniaré to other study sites was evaluated by the statistical rank correlation analysis between the level of *Schistosoma* spp. prevalence and modeled environmental suitability (Table 4). This relationship was not significant in the study site Ouagadougou, which shows that in this region other factors besides environmental suitability modify and thus explain the spatial heterogeneous distribution of disease prevalence (Fig 4).

**Table 4:** Spearman rank correlation coefficients of *Schistosoma* prevalence and modelled environmental suitability. For this analysis, environmental suitability was extracted as mean value from a 5 km buffer zone around the measured prevalence. The corresponding confidence intervals are given in brackets. The columns represent the study sites and the rows represent the habitat variables.

	Ouagadougou	Man	Taabo
**Suitability of habitat stability (HS)**	-0.14 (-0.36, 0.09)	**0.50** * **(0.30, 0.65)**	0.32 (-0.00, 0.58)
**Water temperature suitability (TS)**	-0.08 (-0.30, 0.15)	**0.47** * **(0.27, 0.63)**	-
**Water flow suitability (FS)**	0.04 (-0.19, 0.27)	**0.42** * **(0.21, 0.59)**	0.30 (-0.17, 0.57)
**Water depth suitability (DS)**	-0.23 (-0.44, -0.01)	**0.54** * **(0.36, 0.68)**	-0.15 (-0.45, 0.18)
**Dry season riparian vegetation suitability (dVS)**	0.02 (-0.21, 0.25)	-	-
**Mean vegetation suitability within 200 m buffer zone of water (bVS)**	0.22 (0.00, 0.43)	**0.47** * **(0.27, 0.63)**	0.05 (-0.27, 0.36)
**Stream suitability (StS)**	0.09 (-0.14, 0.32)	**0.50** * **(0.30, 0.65)**	0.25 (-0.08, 0.53)
**Sink suitability (SiS)**	-0.12 (-0.34, 0.11)	0.20 (-0.03, 0.41)	-0.10. (-0.41, 0.23)
**Potential water flow suitability in streams (pFS)**	-0.09 (-0.31, 0.14)	**0.42** * **(0.21, 0.59)**	0.30 (-0.02, 0.56)
**HSI**	-0.12 (-0.34, 0.11)	**0.45** * **(0.25, 0.62)**	**0.57** * **(0.31, 0.75)**

*p < 0.01

In contrast, modeled environmental suitability for the Man region resulted in low suitability derived in the mountainous region in the northern part of this site and high suitability in the lowland of the southern part (Fig 4). In the Man region, areas of high suitability for disease transmission were predominantly represented by the course of rivers. Water buffer zones were largely covered by forest and did not correspond to high environmental suitability due to irrigated agricultural areas as derived in the savannah region of Burkina Faso. Nevertheless, a significant rank correlation to prevalence of schistosomiasis was found for all habitat variables except for sink suitability and for the HSI (Table 4).

Environmental suitability for schistosomiasis transmission in Taabo showed the highest correspondence between the level of environmental suitability and the measured schistosomiasis prevalence with a significant Spearman rank correlation coefficient of 0.57 (Table 4). This positive relation is confirmed by the spatial distribution of high prevalence rates in close proximity to the Bandama River and Lake Taabo, whereas low prevalence rates were predominantly distributed further away from these hotspots of environmental suitability with few exceptions close to the river outflow of Lake Taabo (Fig 4).

## Discussion

In the current study, we developed a new modeling approach, which is based on a multitude of remotely sensed environmental metrics to delineate potential schistosomiasis transmission sites, and allows quantification of suitability for disease-related parasite and snail species. The validation of habitat variables in relation to field measurements and observations showed partial agreement. This validation procedure aimed at identifying strengths and weaknesses of remote sensing environmental metrics to assess environmental suitability for potential schistosomiasis transmission. Lessons learned are offered for discussion.

### Habitat Variables

The high spatial resolution of RapidEye and Landsat 5 TM sensor provided relevant environmental information to detect the small-scale heterogeneity of water bodies in the study sites, except in the Man region, where 6.5 m was still too coarse as water bodies predominantly consisted of small reservoirs and rivers, which, additionally, are often covered by large trees. Water temperature is considered an important habitat variable from an ecological point of view, as temperature is critical for both snail and parasite development. However, remotely sensed water surface temperature provides only to some extent the water temperature that directly impacts snail and parasite development and reproduction. Especially within isolated and shallow water bodies, extreme temperature variations were detected by Fisher and Mustard [95]. The measurement deviation in the range of 10°C between field water temperature and remotely sensed surface temperature might be caused by the inappropriate spatial resolution of the remotely sensed temperature measurement at very small water sites, such as the permanent river water site. Here, the 120 m spatial resolution of the thermal Landsat 5 TM band did not capture the linear structure of the river with width not exceeding 10 m and resulted in mixed pixel information of water and land surface temperature. This results in incorrect surface temperature measurements due to the calibration of surface emissivity for water. At this scale, very high resolution thermal remote sensing data would be more useful to derive water temperature of rivers and streams as shown by Torgersen et al. [96] using air-borne sensors.

Most optical remote sensing data capture visible and near infrared spectral reflectance, and are therefore well designed to measure vegetation coverage. In this study, vegetation was reasonably detected by high-resolution RapidEye data in comparison to field-based estimates of vegetation coverage for rice fields and irrigated crops. It must be noted that vegetation plays a crucial role for characterizing potential schistosomiasis transmission sites also in the submerged area, because submerged vegetation influences dissolved oxygen content of the water body. As a result, snail activity and reproduction are influenced by submerged vegetation [30]. Hyperspectral remote sensing data have demonstrated the ability to detect submerged aquatic vegetation [97–99], but are not yet regularly available as well as for large spatial extents.

The topography derived from the ASTER GDEM provided information on the topographic structure below the detected water level in all water bodies except for the great dam lake in the study site Ziniaré. Therefore, the measurement of slope for water sites often did not correspond to the water surface as intended for this model. This was well documented by strong heterogeneities of the slope measure within a dam lake, which was evident to have a flat surface of stagnant or very slow moving water. Additionally, the temporal dynamic of flow velocity between dry season and wet season as seen for permanent river water sites could not be captured by the single acquisition of a DEM. Nonetheless, suitability of flow velocity showed reasonable predictions for the large dam lake in the Ziniaré study site, and hence, was considered a useful proxy to highlight very flat zones within topographic streams. For large rivers, Kiel et al. [100] successfully derived water flow velocity using Shuttle Radar Topography Mission (SRTM) data. Topographically derived streams were very often not superimposing with the course of the actual river bed visible in high-resolution remote sensing data, which was further documented at the dry river bed test site. However, topographic sinks documented very well the course of river beds and could successfully detect a field measured sink.

### Modeling Environmental Suitability

Environmental suitability for schistosomiasis transmission was modeled based on single habitat variables, which were parameterized by theoretical functions of relative suitability. These functions were derived from different background information, which is demonstrated by the following examples: habitat suitability related to water temperature was derived from laboratory-based measurements [34,35], whereas stream order suitability was characterized from field-based surveys [101] or spatial analysis [87]. The parasite-related water temperature suitability function was directly provided in the literature, whereas snail-related water temperature suitability was interpolated from measurements of snail mortality at given temperatures obtained from the extent literature [34,35]. The thresholds that characterize the range and course of functions given in this study were either cited in the literature (e.g., habitat stability-related suitability), measured within high-resolution remote sensing data (e.g., vegetation coverage), or directly estimated in the field (e.g., water depth). These mixed sources of information can impact the resulting suitability of a habitat variable, because the functions have to be fitted to the modeled location. Due to the lack of appropriate field data on prevalence or fitness of parasites and snails, the parameterization of these functions could not be verified. However, these functions reflect the up-to-date knowledge on the ecology of schistosomiasis parasites and intermediate host snails and provide the basis for location-specific validations. The strengths and weaknesses of selected functions of relative suitability used in this study are discussed in the Supporting Information together with their derivation.

Habitat variables were aggregated to a composite HSI and assessed in relation to reference test sites scored into the classes low, moderate, and high environmental suitability. The model composition was adapted to the prerequisite of schistosomiasis transmission at the study site Ziniaré, and provides a transparent basis to reproduce and adjust the model. The implausible HSI at the seasonal water in dam lake and the dried river bed could be explained by the inappropriate spatial resolution of remotely sensed water temperature and topography. However, a precise identification of key factors determining the suitability of any particular habitat is difficult due to complex interactions [88]. These interactions are considered to some extent through the aggregation of single habitat variables to the HSI. It must be noted that the modeled environmental suitability represents fundamental ecological niches of *S*. *haematobium* and *S*. *mansoni* and *Bulinus* and *Biomphalaria*, which do not necessarily imply that species are abundant at an appropriate site. Parasites and snails can still be absent from apparently suitable habitats, because isolation of individual habitats and (re-)invasion are dictated by chance combinations of factors that permit snail dissemination [88].

### Model Transferability and Public Health Relevance

The transfer of the model from its development site Ziniaré to other selected study sites showed that the specific landscape configuration of dam lakes and irrigated agriculture was highly relevant for potential schistosomiasis transmission in Burkina Faso. However, this was not given in the two study sites of Côte d’Ivoire. In the Taabo setting there is a large man-made lake, but there are no irrigated agricultural sites in close proximity, whilst in the Man area hardly any small dam lakes could be detected, but instead many rivers with dense forests at their watersides. Hence, the 200 m buffer zone around water bodies in Côte d’Ivoire was not considered representative of high environmental suitability given by the index in most areas, which hampered the direct transferability of the model from one geographic setting to another. However, water and potential water accumulation were well represented in all three study sites providing a reasonable basis to monitor potential disease transmission sites in a spatially explicit manner.

The hypothesis that the level of measured prevalence corresponds to the level of environmental suitability for schistosomiasis transmission was confirmed in the Taabo and Man settings, but declined for the study site Ouagadougou. It is clear that suitable environmental conditions provide the prerequisite that transmission of the disease may occur. However, there are factors that influence the relation between environmental suitability and schistosomiasis prevalence, such as local disease intervention measures [5,6], economic development [102], individual disease susceptibility [83,103], and human behavior [104,105].

The spatially explicit information of potential disease transmission sites highlighted in the current paper could provide a new tool for transmission control by guiding public health intervention measures directly to the place where humans become infected. The quantitative information of environmental suitability can support the prioritization of areas for transmission prevention. On the other hand, this model can complement research activities that elaborate on existing differences in disease transmission, occurrence of reservoir hosts, or habitats of parasites and intermediate host snails [7–9].

### Outlook

Remote sensing data have shown the potential to spatially delineate the prerequisite that schistosomiasis transmission may occur through detection of water bodies and sites of potential water accumulation. Additionally, potential transmission sites have been evaluated for their suitability with respect to habitat preferences of disease-related snail and parasite species by means of remote sensing. The model presented in this study can be readily reproduced and adjusted by other experts as new data and information arise. A comprehensive validation of this mechanistic model approach would consist of a direct linkage between derived environmental suitability and field data of parasite- and snail-related fitness.

A predictive map of environmental suitability will support prevention and control measures against schistosomiasis, in line with the recent shift from morbidity to transmission control [7–9]. Despite numerous constraints and limitations of the underlying models of environmental suitability, simple and even untested HSI models were continuously used for different kinds of decision-making procedures [50]. These maps could be useful to target specific locations for initial epidemiologic surveillance or to focus on areas of high reinfection potential for more regular re-treatment [20,106]. The model could provide a useful tool to monitor the development of new hotspots of potential disease transmission, as updated remote sensing data could directly detect changing environmental conditions such as the emergence of new dam lakes in remote areas.

## Supporting Information

S1 Text Derivation of Functions of Relative Suitability. (DOCX)

S2 Text Discussion of Functions of Relative Suitability. (DOCX)

S3 Text Additional References of Supporting Information. (DOCX)
