# Supplementary material for: Modeling and Validation of Environmental Suitability for Schistosomiasis Transmission Using Remote Sensing
Source: PLoS Negl Trop Dis. 2015 Nov 20;9(11):e0004217. doi: 10.1371/journal.pntd.0004217 (PMC4654500; doi:10.1371/journal.pntd.0004217)
Supplement: S1 Text — (DOCX) [file pntd.0004217.s001.docx]

**S1 Text. Derivation of Functions of Relative Suitability**

The prerequisite that schistosomiasis transmission can occur, is the availability of water. With remote sensing data, water bodies were mapped directly from high-resolution RapidEye data. Additionally, the presence of potential water bodies was mapped in an indirect way from topographic information of sinks and drainage lines, where water can potentially accumulate following rainfall or flooding events. The direct measurement of water during dry and rainy season has been further extended with a buffer zone of 200 m. This distance was measured from remote sensing data during the dry season to capture the irrigated agricultural sites, which are directly connected to permanent or seasonal water bodies in the study site Ziniaré. Based on this mask of water and potential water, areas of potential disease transmission have been further refined with quantitative information of snail- and parasite-specific habitat conditions as described by the functions of relative suitability listed in Table 2.

The variable **habitat stability** is defined as length of water persistence in weeks and was derived from multitemporal RapidEye data from the dry and wet season in the year 2010. Water bodies persisting less than 4 weeks for the case of *S. mansoni* and less than 5 weeks for *S. haematobium* were considered unsuitable, respectively [[88](#_ENREF_88)]. If water in a snail habitat remains for longer than 6 or 7 weeks, the habitat stability was considered suitable for transmission of *S. mansoni* or *S. haematobium*, respectively [[88](#_ENREF_88)]. The relative suitability function that connects the unsuitable and suitable state of habitat stability results from a linear interpolation between the measured values.

The relation between **water temperature** and the length of the prepatent period of parasites was defined as response function for *S. mansoni* by [Pflüger [34](#_ENREF_34)] and for *S. haematobium* by [Pflüger et al. [35](#_ENREF_35)]. These species response functions were directly scaled to functions of relative suitability of temperature, in which the shortest prepatent period corresponded with highest suitability for parasite development. Additionally, the relation between water temperature and snail mortality was investigated for snails collected in the field and observed under laboratory conditions. *Bio. glabrata*, the intermediate host snails of *S. mansoni* showed a mortality rate close to 100% at experimental water temperatures below 16°C and above 36°C [[34](#_ENREF_34)]. The mortality thresholds of *Bu. truncatus* snails, the intermediate host snails of *S. haematobium* were at temperatures below 17°C or above 33°C [[35](#_ENREF_35)]. The laboratory-based measurements of snail mortality [[34](#_ENREF_34),[35](#_ENREF_35)] were interpolated based on a second order polynomial function and resulted respective functional relations for *Bio. glabrata* and *Bu. truncatus* snails.

A critical value of **water flow velocity** was established at 0.3 m/s [[86](#_ENREF_86)]. In faster flowing water snails become dislodged and the suitability of the habitat drops immediately. The flow velocity of water $V$ was compared with slopes derived from topographic remote sensing data using the Manning´s velocity equation [[85](#_ENREF_85)]

| $V= \frac{1.5* R^{0.66}* S^{0.5}}{n}$ |
| --- |

where *R* is the hydraulic radius, *S* is the line slope and *n* is the Manning´s roughness coefficient. Within the visited test site around Ziniaré in Burkina Faso, the threshold of 0.3 m/s could be approximated to a slope of 0.00014 degrees. This calculation is based on the assumption that the majority of river beds are gravelled earth channels with some vegetation growth, which are represented by 0.025 for n [[85](#_ENREF_85)]. *R* is equal to the cross-sectional area of flow divided by the wetted perimeter [[107](#_ENREF_107)] and was approximated for this calculation to be 0.7m. This parameter could not be adjusted to the heterogeneous river beds in the study site, because the 30m spatial resolution of topographic data from the ASTER GDEM could not depict this small-scale information. Therefore, a mean width and depth of river beds was assumed to be 5 m and 2 m, respectively. The relative suitability **function of water flow velocity** was derived from linear interpolation between the minimum, the suitability threshold, and the maximum. It was assumed that the relative suitability decreases strongly towards the derived threshold slope of 0.00014 degrees corresponding to a relative suitability of 0.2 and levels out with this general low suitability towards 0.

The relative suitability of **water depth** is expressed by the proxy measurement of Euclidean distance from the shoreline, which was calculated from the polyline boundary of the water masks from the dry and wet season. Intermediate host snails of schistosomiasis are primarily distributed in shallow water at the margins of their habitat [[90](#_ENREF_90)]. This information was translated into a decreasing suitability derived from linear interpolation between shoreline and a distance of 200 m inside the water. The threshold was estimated based on the slope to distance ratio between the maximum water level and the current one during the dry season around the visited dam lakes. For this calculation, the threshold was adjusted to 210 m to multiply the 30 m pixel resolution of the satellite data employed in the current study. At distances greater than 2 km from the shoreline to the center of the water body, no suitability for snails transmitting schistosomiasis was assumed. This threshold was extracted from the extent of the greatest water body in the study site.

The relative suitability based on **vegetation coverage** was measured within a 200 m buffer area along detected water sites. Higher availability of vegetation positively conditions the habitat for freshwater snails in terms of food supply, surfaces to crawl and deposit egg masses or with respect to the content of dissolved oxygen in water [[30](#_ENREF_30)]. The remote sensing approach used in this study does not measure submerged vegetation, however, the buffer zone considers the potential vegetation input into the water body. In this model, the theoretical function of relative suitability with respect to vegetation coverage was derived to detect irrigated agricultural sites visited during the field trip, which corresponded to an average NDVI threshold around 0.3 in dry season RapidEye imagery. Based on this threshold, the sub-priority function of vegetation coverage was a result of linear interpolation between unsuitable conditions for NDVI values smaller or equal 0 and suitable conditions at values of 0.3 and above.

**Stream order** resulted in hierarchical levels ranging from order 1 to 7. According to the study of [Beck-Wörner et al. [87](#_ENREF_87)], the relative suitability function for stream order was estimated from a linear interpolation between the maximum stream order of highest suitability and the minimum stream order of lowest suitability.

Sink depth resulted in a maximum depth of 222 m for the study area. The function of relative suitability of sink depth was calculated from linear interpolation between the lowest sink depth of 1 m and the aforementioned maximum sink depth of 222 m.
