# Supplementary material for: Modeling and Validation of Environmental Suitability for Schistosomiasis Transmission Using Remote Sensing
Source: PLoS Negl Trop Dis. 2015 Nov 20;9(11):e0004217. doi: 10.1371/journal.pntd.0004217 (PMC4654500; doi:10.1371/journal.pntd.0004217)
Supplement: S2 Text — (DOCX) [file pntd.0004217.s002.docx]

S2 Text. Discussion of Functions of Relative Suitability.

The water flow velocity based suitability has been parameterised based on the given suitability threshold in relation to snail prevalence and the defined relation between the remote sensing measurement of slope and water flow velocity. This distinct relation can be exposed to error sources that need to be considered with respect to input data, namely the inconsistent remote sensing measurement of slope at water sites as well as the rough estimation of several variables necessary to compute the Manning’s equation at only few locations in the field. To receive more accurate information on flow velocity, it would be necessary to derive these input parameters specifically at location, which is not feasible with the 30 m resolution ASTER GDEM within the small scale heterogeneity of the terrain as experienced in this study site. Nevertheless, the water flow velocity based suitability showed reasonable values for large stagnant water bodies such as the large dam lake in the Ziniaré study site and was considered a useful proxy to highlight very flat zones within topographic streams.

The theoretical function to parameterise the water depth-related suitability was based on subjective thresholds, which were not validated with respect to its relation to water depth but were solely estimated during the field visit at the seasonally dried areas of a dam lake boundary. The thresholds of 210 m and 2 km need to be adjusted for rivers, which have a steep gradient towards the main current line and are often narrow. Nevertheless, the distance from water level was considered a useful proxy with respect to dam lakes and provides reasonable information that suitability of schistosomiasis transmission is highest in the very flat littoral zones of a water body as indicated by the [WHO [90](#_ENREF_90)]. Other studies found that if water depth measurements were provided, spectral reflectance of airborne multispectral imagery [[108](#_ENREF_108),[109](#_ENREF_109)], Landsat 5 TM data [[110](#_ENREF_110)] or data from the IRS-LISS sensor [[111](#_ENREF_111)] were successfully used to estimate water depth based on regression models.

The theoretical function of topographic sink based suitablity represents the logical assumption that the deeper sinks are the longer water persists, which resulted in higher suitability for schistosomiasis transmission. This function derived from linear interpolation between minimum and maximum sink depth is certainly modified by the given sink depth thresholds within the area of interest. A validation of sink depth and its relation to water holding capacities would require additional field data considering precipitation and soil drainage. With respect to precipitation, rainfall measurements derived from the TRMM data were not considered useful for this study site due to the large scale mismatch between a 30 m resolution suitability image and the TRMM resolution of 28 km. This scale mismatch was similar to the soil characteristics provided by the [FAO et al. [112](#_ENREF_112)].
