# Supplementary material for: Modeling and Validation of Environmental Suitability for Schistosomiasis Transmission Using Remote Sensing
Source: PLoS Negl Trop Dis. 2015 Nov 20;9(11):e0004217. doi: 10.1371/journal.pntd.0004217 (PMC4654500; doi:10.1371/journal.pntd.0004217)
Supplement: S3 Text — (DOCX) [file pntd.0004217.s003.docx]

**S3 Text:** **Additional References of Supporting Information**

107. Arcement GJ (1989) Guide for selecting Manning's roughness coefficients for natural channels and flood plains. US Department of the Interior - United States Geological Survey.

108. Gilvear DJ, Waters TM, Milner AM (1995) Image analysis of aerial photography to quantify changes in channel morphology and instream habitat following placer mining in interior Alaska. Freshwater Biol 34: 389-398.

109. Winterbottom SJ, Gilvear DJ (1997) Quantification of channel bed morphology in gravel-bed rivers using airborne multispectral imagery and aerial photography. Regul River 13: 489-499.

110. Bierwirth PN, Lee TJ, Burne RV (1993) Shallow seam floor reflectance and water depth derived by unmixing multispectral imagery. Photogramm Eng Remote Sens 59: 331-338.

111. Kumar KV, Palit A, Bhan SK (1997) Cover bathymetric mapping in Rupnarayan-Hooghly river confluence using Indian remote sensing satellite data. Int J Remote Sens 18: 2269-2270.

112. FAO, IIASA, ISRIC, ISS-CAS, JRC (2012) Harmonized world soil database (version 1.2). Rome, Italy & Laxenburg, Austria: FAO & IIASA.
